# Depression im Alter und Frailty – epidemiologische, klinische und neurobiologische Zusammenhänge

**DOI:** 10.1007/s00115-023-01444-0

**Published:** 2023-02-17

**Authors:** M. S. Depping, L. Köhler-Ipek, P. Ullrich, K. Hauer, R. C. Wolf

**Affiliations:** 1 Klinik für Allgemeine Psychiatrie, Zentrum für Psychosoziale Medizin, Universitätsklinikum Heidelberg, Voßstr. 4, 69115 Heidelberg, Deutschland; 2 Geriatrisches Zentrum an der Medizinischen Fakultät der Universität Heidelberg, Agaplesion Bethanien Krankenhaus Heidelberg, Rohrbacher Str. 149, 69126 Heidelberg, Deutschland

**Keywords:** Gerontopsychiatrie, Biologische Alterung, Belohnungsverarbeitung, Bewegungsinitiierung, Apathie, Geriatric psychiatry, Biological aging, Reward processing, Movement initiation, Apathy

## Abstract

**Hintergrund:**

Depression ist beim alten Menschen die häufigste psychische Störung und wird durch geriatrietypische Morbidität beeinflusst. Die Komorbidität mit „Frailty“ ist besonders relevant. Frailty ist von zentraler Bedeutung in der modernen Altersmedizin und zeigt die belastungsabhängige Vulnerabilität eines alten Menschen sowie sein erhöhtes Risiko von Behinderung, Hospitalisierung und Tod an. Es kommt der Diagnostik und Behandlung von Depression im Alter zugute, sich mit den Zusammenhängen mit Frailty auseinanderzusetzen, auch auf neurobiologischer Ebene.

**Ziel der Arbeit:**

Dieses narrative Review gibt einen Überblick über die Komorbidität von Depression im Alter und Frailty, mit einem Schwerpunkt auf neurowissenschaftlichen Erkenntnissen, die anhand des Research-Domain-Criteria(RDoC)-Ansatzes systematisiert werden.

**Ergebnisse:**

Frailty findet sich komorbid bei mehr als einem Drittel der Patienten mit Depression im Alter, was mit kritischen Depressionsverläufen und mit schlechterer Wirksamkeit und Verträglichkeit antidepressiver Medikation verbunden ist. Depression und Frailty teilen motivationale und psychomotorische Merkmale, speziell Antriebsminderung, erhöhte Erschöpfbarkeit und verringerte körperliche Aktivität. Bei Frailty sind funktionelle Veränderungen in bewegungsvorbereitenden neuronalen Arealen mit motorischen Leistungseinschränkungen assoziiert. Bei Depression im Alter mit Apathie finden sich abnorme Struktur und veränderte funktionelle Konnektivität des Belohnungs- und des Salienznetzwerks, außerdem veränderte funktionelle Konnektivität dieser Netzwerke mit prämotorischen Arealen.

**Diskussion:**

Es ist prognostisch und therapeutisch relevant, Frailty bei Alterspatienten mit Depression zu erkennen. Die (Weiter‑)Entwicklung und Individualisierung von Therapien für diese vulnerable Patientengruppe wird auch davon profitieren, sich auf neuronale Mechanismen der Komorbidität zu beziehen.

## Abstract (en)

**Background:**

Depression is the most common mental disorder in older adults and is influenced by age-related processes. Frailty is a well-established clinical expression of ageing that implies a state of increased vulnerability to stressor events as well as increased risks of disability, hospitalization and death. Neurobiological findings will disentangle the comorbidity of frailty and depression and may inform future management of depression in old age.

**Objective:**

This narrative review provides an overview of the comorbidity of late-life depression and frailty, with a focus on neuroscientific findings that are organized within the research domain criteria (RDoC) framework.

**Results:**

More than one third of old people with depression are affected by frailty, which results in more chronic depression and in poorer efficacy and tolerability of antidepressant medication. Depression and frailty share motivational and psychomotor characteristics, particularly apathy, decreased physical activity and fatigue. In patients with frailty, altered activity of the supplementary motor cortex is associated with motor performance deficits. Patients with late-life depression and apathy are characterized by abnormal structure and altered functional connectivity of the reward network and the salience network, along with altered functional connectivity of these networks with premotor brain areas.

**Conclusion:**

Identifying frailty in older adults with depression is relevant for prognostic assessment and treatment. A better understanding of the neuronal mechanisms of comorbidity will provide potential targets for future personalized therapeutic interventions.

Als Depression im Alter wird eine erstmalig ab dem 65. Lebensjahr auftretende depressive Symptomatik bezeichnet, die die gängigen, altersübergreifenden Diagnosekriterien für depressive Störungen erfüllt. Das Depressionsrisiko im Alter wird durch altersabhängige Erkrankungen beeinflusst [3]. Während die pathogenetische Rolle zerebraler Mikroangiopathie in den letzten 25 Jahren intensiv beforscht wurde (was zur vaskulären Depressionshypothese geführt hat [3]), ist das für die Geriatrie zentrale „Frailty“-Syndrom erst in neueren Untersuchungen zur Depression im Alter in den Blick genommen worden.

## Hintergrund

Bei einem Patienten mit Depression im Alter deutet es auf eine mögliche Komorbidität mit „Frailty“ hin, wenn psychomotorische und motivationale Symptome stark ausgeprägt sind, speziell Antriebsminderung, erhöhte Erschöpfbarkeit und verringerte körperliche Aktivität [26]. Bestätigt ein strukturiertes Assessment Frailty, sind ein chronischer Depressionsverlauf [25] und ein Nichtansprechen auf Therapie mit Serotoninwiederaufnahmehemmern (SSRI; [6]) wahrscheinlicher, ebenso sind die Risiken von Alltagsbeeinträchtigung, Pflegeheimeinweisung und Sterblichkeit erhöht [40]. Die Komorbidität von Depression im Alter und Frailty ist ein häufiges Phänomen, das mehr als ein Drittel der Alterspatienten mit Depression betrifft [40]. Auch angesichts der hohen Symptomübereinstimmung zwischen Frailty und Depression (besonders, wenn sie durch Apathie gekennzeichnet ist; [26]) stellt sich die Frage, in welchen Zusammenhängen Depressionen im Alter mit dem biologischen Altersphänomen „Frailty“ stehen. Solche Erkenntnisse über altersspezifische Pathomechanismen werden die Behandlung von Depressionen im höheren Lebensalter erweitern. Hierzu besteht ein dringender Bedarf, sind doch altersspezifische Strategien in der Depressionstherapie bisher unterrepräsentiert [10].

Dieses narrative Review gibt eine Übersicht über epidemiologische, klinische und neurobiologische Zusammenhänge von Depression im Alter und Frailty. Potenzielle Gemeinsamkeiten auf der Ebene neuronaler Funktionssysteme werden auf den Research-Domain-Criteria(RDoC)-Ansatz bezogen [16, 21], wodurch sich auch therapeutische Ansätze systematisieren lassen.

## Bedeutung des Frailty-Konzeptes

Frailty ist ein modernes, für die Altersmedizin zentrales Konstrukt. Es stellt einen Versuch dar, das biologische Alter eines älteren Menschen zu erfassen [12]. Frailty liegt ein altersbedingter Funktionsverlust verschiedener physiologischer Systeme zugrunde, was zu verminderten physiologischen Reserven und zu erhöhter Vulnerabilität gegenüber verschiedensten Stressoren führt (z. B. Krankheiten, soziale Umgebungsfaktoren; [12]). Für Betroffene bedeutet dies ein erhöhtes Risiko von Behinderung, Hospitalisierung und Tod [12]. Die am besten etablierte Definition von Frailty verwendet 5 Kriterien: verminderte Kraft, verminderte Gehgeschwindigkeit, verringerte körperliche Aktivität, erhöhte Erschöpfbarkeit und/oder ungewollter Gewichtsverlust. Sind mindestens 3 der 5 Kriterien in beliebiger Kombination erfüllt, wird von Frailty ausgegangen (Phänotyp nach Fried; [14]). Frailty hat eine hohe prognostische Aussagekraft, die über eine alleinige Zustandsbeschreibung als „gebrechlich“ hinausgeht [12].

## Epidemiologie und Klinik von Depression im Alter mit komorbider Frailty

Das Risiko, eine Depression zu entwickeln, ist bei Alterspatienten mit Frailty gegenüber robusten Gleichaltrigen um das Vierfache erhöht [40]. Frailty begünstigt chronische Depressionsverläufe [25].

Geringe körperliche Aktivität, Antriebsminderung und erhöhte Erschöpfbarkeit sind geteilte Merkmale, in denen sich Frailty und Depression auf bemerkenswerte Weise ähneln [26]. In den diagnostischen Instrumenten von Frailty bzw. Depression werden sie z. T. identisch erfragt [42]. In den meisten der verfügbaren Frailty-Assessments kann die diagnostische Schwelle für Frailty alleine durch eine Kombination von Merkmalen erreicht werden, die Depressionskriterien gemäß DSM‑5 darstellen [42]. Dies hat bis zur Frage geführt, ob Frailty und Depression fächerspezifische Operationalisierungen eines gleichen Zustands sein könnten. Epidemiologische Untersuchungen mit Faktorenanalysen weisen diese Hypothese zwar zurück, betonen aber die hohe Assoziation von Frailty und Depression im Alter [26].

## Neuronale Dysfunktion bei Frailty und Depression im Alter

Gehirnerkrankungen können zu Frailty beitragen, z. B. neurodegenerative Erkrankungen [9]. Weitgehend unbekannt ist hingegen, ob und wie Frailty mit der Integrität von Gehirnstruktur und -funktion zusammenhängt, wenn Patienten nicht an einer neurologischen Erkrankung leiden. Einige neuere Pilotstudien haben Frailty-Patienten mit Magnetresonanztomographie (MRT) unter Anwendung moderner Datenanalyseverfahren untersucht: Subtile Hirnstrukturveränderungen liegen bei Frailty-Patienten verteilt über das Gehirn vor [11, 27, 44]. Veränderte neuronale Bewegungsvorbereitung im supplementär-motorischen Areal (SMA) und im Prä-SMA, ausgedrückt in verminderter funktioneller Konnektivität innerhalb der funktionellen (Prä‑)SMA-Netzwerke, ist mit dem Frailty-Status und mit motorischen Leistungseinschränkungen verbunden [23, 24]. Auch die Integrität nichtmotorischer funktioneller Netzwerke ist bei Frailty-Patienten verändert [41].

Die epidemiologischen und klinischen Zusammenhänge von Frailty und Depression im Alter motivieren zu transdiagnostischen Fragestellungen, die auf breite neurowissenschaftliche Vorbefunde zur Depression Bezug nehmen können:

1. Teilen sich Frailty und Depression auch neuropathologische Merkmale?
2. Welchen Einfluss hat Frailty auf die für Depression bedeutsamen neuronalen Systeme, kommt es z. B. zu neuronalen Wechselwirkungen bei Komorbidität?

Motivationale bzw. motorische Funktionen bilden 2 von 6 Domänen des Research-Domain-Criteria(RDoC)-Ansatzes. Der RDoC-Ansatz formuliert eine neue Forschungssystematik für die biologische Psychiatrie, die sich von traditioneller psychiatrischer Klassifikation löst und stattdessen neuropsychiatrische Symptome systematisch mit neurobiologischen Funktionssystemen in Zusammenhang bringt [16, 21]. Transdiagnostische Untersuchungen sind nicht nur exzellent mit dem RDoC-Ansatz vereinbar, sondern werden ausdrücklich von dessen Initiatoren gefordert [16, 21].

### Positive Valenzsysteme

**Figure 1:**
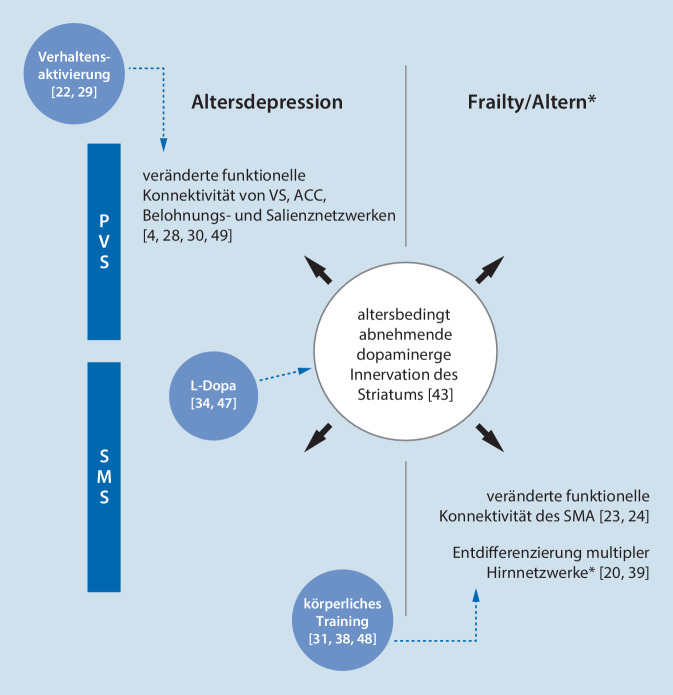

Reduzierter Handlungsantrieb und vermindertes zielbezogenes Verhalten sind motivationale Analyseeinheiten der RDoC-Domäne Positive Valenzsysteme (PVS). Sie werden mit beeinträchtigter Funktion des neuronalen Belohnungssystems in Zusammenhang gebracht [35] und wurden vielfach bei Depressionspatienten untersucht. Ein Mangel an annäherndem, positiv motiviertem Verhalten beruht demnach auf vermindertem neuronalem Ansprechen auf belohnungsanzeigende Stimuli [15], auf eingeschränktem Erlernen neuer Anreizwerte [32] sowie auf dysfunktionaler Entscheidungsfindung, wenn antizipierte Belohnung und dafür aufzubringende Anstrengung gegeneinander abzuwägen sind [35]. Reduzierte Motivation bei Patienten mit Depression ist auch mit veränderter funktioneller Konnektivität des Belohnungssystems mit anderen neuronalen Netzwerken assoziiert [33]. Speziell bei Alterspatienten mit Depression und vordergründiger Apathie wurden Funktionsabweichungen von ventralem Striatum (VS) und anteriorem Zingulum (ACC) sowie veränderte funktionelle Konnektivität in den zugehörigen Belohnungs- und Salienznetzwerken aufgezeigt (Abb. 1; [4, 28, 30, 49]). Auch wurde veränderte funktionelle Konnektivität dieser Systeme mit dem prämotorischen Kortex beschrieben [28].

Motivationale Prozesse, speziell belohnungsabhängiges Lernen, unterliegen phasischer dopaminerger Innervation des VS [35]. Altersbedingt nehmen dopaminerge Neurone im Mesenzephalon sowie Dopamintransporter und dopaminerge Rezeptoren im Striatum ab (Abb. 1; [43]). Hierunter sind bei älteren Menschen belohnungsabhängiges Lernen und anstrengungsabhängiges Entscheidungsverhalten verändert [43], was sowohl für die Entstehung von Depression im Alter mit Apathie als auch von Frailty relevant sein könnte [8, 43].

### Sensomotorische Systeme

Eingeschränktes Bewegungsverhalten bzw. körperliche Inaktivität werden von der RDoC-Domäne Sensomotorische Systeme (SMS) abgebildet. Die Domäne unterscheidet die motorischen Teilprozesse Handlungsplanung und -auswahl, Bewegungsinitiation, -ausführung und -beendigung sowie sensomotorische Dynamiken [16] und betont im Hinblick auf zielgerichtetes Handeln das Zusammenspiel mit motivationalen Prozessen [16]. Bei Depression stehen MRT-Untersuchungen zu motorischen Funktionen erst in den Anfängen [46]. Unter dem Einfluss motivationaler Defizite könnten beispielsweise die neuronale Bewegungsvorbereitung und -initiierung im SMA verändert sein [1] und körperliche Inaktivität bei Depressionspatienten unterhalten werden [5]. Funktionsabweichungen des SMA werden als Korrelat motorischer Depressionsphänomene diskutiert [46].

Bei Frailty-Patienten wurden motorische Leistungseinschränkungen bisher in Pilotstudien mit veränderter funktioneller Konnektivität des SMA in Verbindung gebracht (Abb. 1; [23, 24]). Lassen motorische Fähigkeiten im Alter nach (was langfristig unvermeidbar ist, aber bei Frailty beschleunigt abläuft), geht dies unter experimenteller Anforderung mit veränderter neuronaler Aktivierung in motorischen Gehirnarealen im Vergleich zu jüngeren Erwachsenen einher [37]. Gleichzeitig ist die funktionelle Konnektivität zwischen motorischen und nichtmotorischen Hirnnetzwerken mit zunehmendem Alter erhöht, zugunsten verminderter funktioneller Konnektivität innerhalb der Netzwerke [20]. Diese altersbedingte Entdifferenzierung von Hirnnetzwerken (Abb. 1) ist nicht nur mit der motorischen Leistungsfähigkeit, sondern auch mit dem körperlichen Aktivitätsniveau im Alltag assoziiert [39]. Frailty ist Ausdruck beschleunigter Alterung [12] und verringerte körperliche Aktivität ist ein Kardinalsymptom von Frailty [14], aber die Übertragbarkeit dieser neuronalen Alterungsbefunde auf Frailty-Patienten noch unklar.

Der Erwerb von Handlungsroutinen und die situationsgerechte Regulation von Bewegungen, v. a. hinsichtlich Geschwindigkeit und Umfang, sind von dopaminerger Transmission in den Basalganglien abhängig [13]. Die oben beschriebenen Alterungsprozesse im dopaminergen System (Abb. 1) resultieren auch in verminderter Gehgeschwindigkeit [13], was pathogenetisch für Frailty und für Depression im Alter bedeutsam sein könnte [8, 34, 43].

## Therapie

Alterspatienten mit Depression und komorbider Frailty profitieren nur begrenzt von SSRI [6]. Bewegungs- und behaviorale Interventionen, dopaminerge Medikation und ggf. transkranielle Magnetstimulation (TMS) könnten stattdessen geeignet sein, die motivationalen und motorischen Funktionseinschränkungen in dieser Patientengruppe zu mildern. Für diese Verfahren liegen für Patienten mit Depression oder mit Frailty z. T. hochwertige Wirksamkeitsbelege vor [10, 28, 30, 35], allerdings noch keine Untersuchungen bei komorbid Erkrankten.

Körperliches Training ist der am besten untersuchte Ansatz, um Frailty vorzubeugen oder die Ausprägung von Frailty zu minimieren [31]. Bei Depressionspatienten zeigen Bewegungsinterventionen moderate bis große Effektstärken [36]. Körperliches Training beeinflusst die strukturelle und funktionelle Organisation von Hirnnetzwerken [48] und wirkt möglicherweise altersabhängigen Veränderungen in diesen Systemen entgegen (Abb. 1; [38]).

Verhaltensaktivierung ist der wichtigste behaviorale Ansatz zur Depressionsbehandlung. Dabei erhöht ein Depressionspatient systematisch gesunde Verhaltensweisen und die Rate an positiven Verstärkern für dieses Verhalten, wobei körperliche Aktivität oder Training ein häufiger (aber nicht notwendiger) Bestandteil sind. Die antidepressive Wirksamkeit von Verhaltensaktivierung ist gut belegt und hoch, auch bei Alterspatienten [29]. Auf neuronaler Ebene könnte Verhaltensaktivierung die funktionelle Integrität des Belohnungssystems verbessern (Abb. 1; [22]).

L‑Dopa oder Dopaminagonisten sind zur antidepressiven Augmentation bei jüngeren Depressionspatienten unwirksam [10]. Bei Alterspatienten mit Depression verbessert die Gabe von L‑Dopa allerdings eine verlangsamte Gehgeschwindigkeit, mit möglicher antidepressiver Wirkung (Abb. 1; [34]). Ob die Gabe von L‑Dopa auch die neuronale Belohnungsverarbeitung bei Depressionspatienten günstig beeinflussen kann, bleibt noch unklar [47].

Transkranielle Magnetstimulation (TMS) des linken dorsolateralen präfrontalen Kortex ist eine evidenzbasierte Behandlungsoption, die Patienten mit pharmakoresistenter Depression angeboten werden kann [10]. Auch für depressive Alterspatienten gibt es Wirksamkeitsbelege [19]. Pilotstudien haben außerdem TMS des SMA erfolgreich angewandt, um psychomotorische Verlangsamung bei Depressionspatienten abzuschwächen [45]. Ob TMS zur Behandlung von Patienten mit Depression im Alter und komorbider Frailty beitragen kann, bleibt experimentell zu prüfen.

## Ausblick

Der in den letzten 25 Jahren am häufigsten diskutierte Subtyp von Depressionen im Alter ist die „vaskuläre Depression“, wobei bis heute kontrovers bleibt, wie mikrovaskuläre Marklagerläsionen, emotions- und motivationsrelevante Hirnnetzwerke sowie Depression zusammenhängen [2, 17]. Auch zwischen mikrovaskulärer Läsionslast und der Ausprägung von Frailty wurden widersprüchliche Assoziationen berichtet [7, 18]. Depressionen im Alter mit komorbider Frailty lassen sich bisher nur unzureichend in Bezug zur vaskulären Depressionshypothese setzen.

Neuere Forschungserkenntnisse unterstreichen die epidemiologische und klinische Bedeutung von Frailty bei Alterspatienten mit Depression. Sie deuten zudem pathophysiologische Wege an, auf denen Frailty zur Entstehung und Aufrechterhaltung von Depression beim älteren Menschen beitragen könnte.

Künftige Studien, die definierte motivationale und motorische Teilprozesse bei Komorbidität von Depression im Alter und Frailty untersuchen, werden Erkrankungsmechanismen aufschlüsseln, den Beitrag von biologischer Alterung zu Depressionen im hohen Lebensalter präzisieren und letztlich die Behandlung dieser vulnerablen Patientengruppe verbessern.

## Fazit für die Praxis

- Die Beurteilung von Frailty unterstützt die Behandlung von Depression im Alter bei der Therapieauswahl, der Verlaufs- und Prognoseabschätzung sowie der Versorgungsplanung.
- Frailty kann durch einfach anzuwendende Instrumente identifiziert werden, z. B. die Klinische Frailty-Skala (CFS). Zur Beurteilung von Depressivität bei Patienten mit Frailty sind Instrumente von Vorteil, die nur wenige oder keine körperlichen Depressionssymptome beinhalten, beispielsweise die Geriatrische Depressionsskala (GDS).
- Frailty und Depression im Alter gehen mit Funktionsstörungen in definierten Hirnnetzwerken einher, die in Zukunft zur Verlaufs- und Therapieprädiktion herangezogen werden könnten.
- Alterspatienten mit Depression und Frailty profitieren von Verhaltensaktivierung und körperlichem Training. Der Nutzen von Psychopharmakotherapie oder Hirnstimulationsverfahren ist für diese vulnerable Patientengruppe noch ungewiss.
